# Integrated Modelling of Cell Responses after Irradiation for DNA-Targeted Effects and Non-Targeted Effects

**DOI:** 10.1038/s41598-018-23202-y

**Published:** 2018-03-19

**Authors:** Yusuke Matsuya, Kohei Sasaki, Yuji Yoshii, Go Okuyama, Hiroyuki Date

**Affiliations:** 1 Graduate School of Health Sciences, Hokkaido University, Kita-12, Nishi-5, Kita-ku, Sapporo 060-0812 Japan; 2 Faculty of Health Sciences, Hokkaido University of Science, Maeda 7-15, Teine-ku, Sapporo 006-8585 Japan; 3 Biological Research, Education and Instrumentation Center, Sapporo Medical University, Minami-1, Nichi-17, Chuo-ku, Sapporo 060-8556 Japan; 4 Faculty of Health Sciences, Hokkaido University, Kita-12, Nishi-5, Kita-ku, Sapporo 060-0812 Japan

## Abstract

Intercellular communication after ionizing radiation exposure, so-called non-targeted effects (NTEs), reduces cell survival. Here we describe an integrated cell-killing model considering NTEs and DNA damage along radiation particle tracks, known as DNA-targeted effects (TEs) based on repair kinetics of DNA damage. The proposed model was applied to a series of experimental data, i.e., signal concentration, DNA damage kinetics, cell survival curve and medium transfer bystander effects (MTBEs). To reproduce the experimental data, the model considers the following assumptions: (i) the linear-quadratic (LQ) function as absorbed dose to express the hit probability to emit cell-killing signals, (ii) the potentially repair of DNA lesions induced by NTEs, and (iii) lower efficiency of repair for the damage in NTEs than that in TEs. By comparing the model results with experimental data, we found that signal-induced DNA damage and lower repair efficiency in non-hit cells are responsible for NTE-related repair kinetics of DNA damage, cell survival curve with low-dose hyper-radiosensitivity (HRS) and MTBEs. From the standpoint of modelling, the integrated cell-killing model with the LQ relation and a different repair function for NTEs provide a reasonable signal-emission probability and a new estimation of low-dose HRS linked to DNA repair efficiency.

## Introduction

Radiosensitivity of cells is affected by not only targeted effects (TEs)^1^ but also non-targeted effects (NTEs)^2–4^. The target theory is based on the idea that hits by radiation make sensitive targets in DNA inactivated and lead to the reduction of cell viability^5^, which may be explained by the number of DNA lesions induced along ionizing radiation particles^1,5^. After irradiation, broken ends of DNA are mostly rejoined by DNA repair functions^6,7^, but a few lethal lesions with chromosome aberrations such as dicentric and ring chromosomes remain, which leads to cell death. Cells without direct hits by radiation are also likely to show the same behavior as TEs, such as abnormal chromosome damage and mutations. These are called NTEs or radiation-induced bystander effects (RIBEs), or in some cases low-dose hyper radio-sensitivity (HRS)^8,9^. NTEs have been interpreted as a consequence of intercellular communication with cell-killing signals^8^. However, these effects remain to be elucidated in detail, particularly at low-dose exposure.

While the mechanisms that induce low-dose HRS are still under investigation, clues are being obtained from biological experiments and theoretical analyses. After irradiation, cell-killing signals are emitted from the radiation hit cells. According to investigations by Stewart *et al*.^10^ and Liu *et al*.^11^, the size of the target to release the signals is on the micrometer order, i.e., 1.0–2.0 μm diameter, which may be related to target sensitivity^12^. The target may also be linked to mitochondria^13^. There are several types of signals, such as cytokines including interleukin 6 (IL-6), calcium, reactive oxygen species (ROS), nitric oxide (NO) and so on^14–21^. These signals are assumed to transfer from hit cells to the non-hit cells via gap junctions or culture medium^22^. The intercellular signaling can induce DNA damage^23^ which may be repaired^24^. However, the damage in non-hit cells sometimes persists for a prolonged time^25^, suggesting that the disorder of DNA repair efficiency may have occurred in non-hit cells^26–28^. Thus, the damage induction and its repair process in the non-hit cells are presumably different from those in the irradiated cells^29^. From the viewpoint of the repair induction system against DNA damage, currently it has been interpreted that an increased radioresistance (IRR) emerges if the repair capacity overcomes the low-dose HRS in a dose up to 0.3 Gy^30^.

TEs on cell survival considering track structure of radiation were successfully modeled by Hawkins, calling it the microdosimetric-kinetic (MK) model^31,32^, which considers microdosimetry^33,34^ and sub-lethal damage (SLD) repair during dose-delivery^32,35,36^. On the contrary, there are several formulations to quantify low-dose HRS and intercellular signaling^30,37–43^. Among them, temporal characteristic of cell-killing signals has been modeled by Kundrát *et al*.^41^, and a stochastic model of signal-induced mutation and cell death was proposed by McMahon *et al*.^40^. However, there is no model analysis considering the kinetics of signal-induced DNA lesions. Thus, our interest was directed to the development of a biophysical model which can evaluate not only the cell survival in TEs and NTEs but also damage kinetics associated with DNA repair in non-hit cells. To our knowledge, this is the first model estimation for a relation between shape of low-dose HRS and DNA repair function in non-hit cells.

In this study, we used an integrated cell-killing model which includes cell responses such as cell survival and DNA damage kinetics in TEs and NTEs, hereafter calling it the integrated MK (IMK) model. By applying the model to reference data of intercellular signals, DNA damage kinetics and surviving fraction after irradiation, our model estimation finally shows that the degree of repair efficiency in non-hit cells is a main factor responsible for modifying low-dose HRS in cell survival curves.

## Model Overview- Targeted Effects

**Figure 1:**
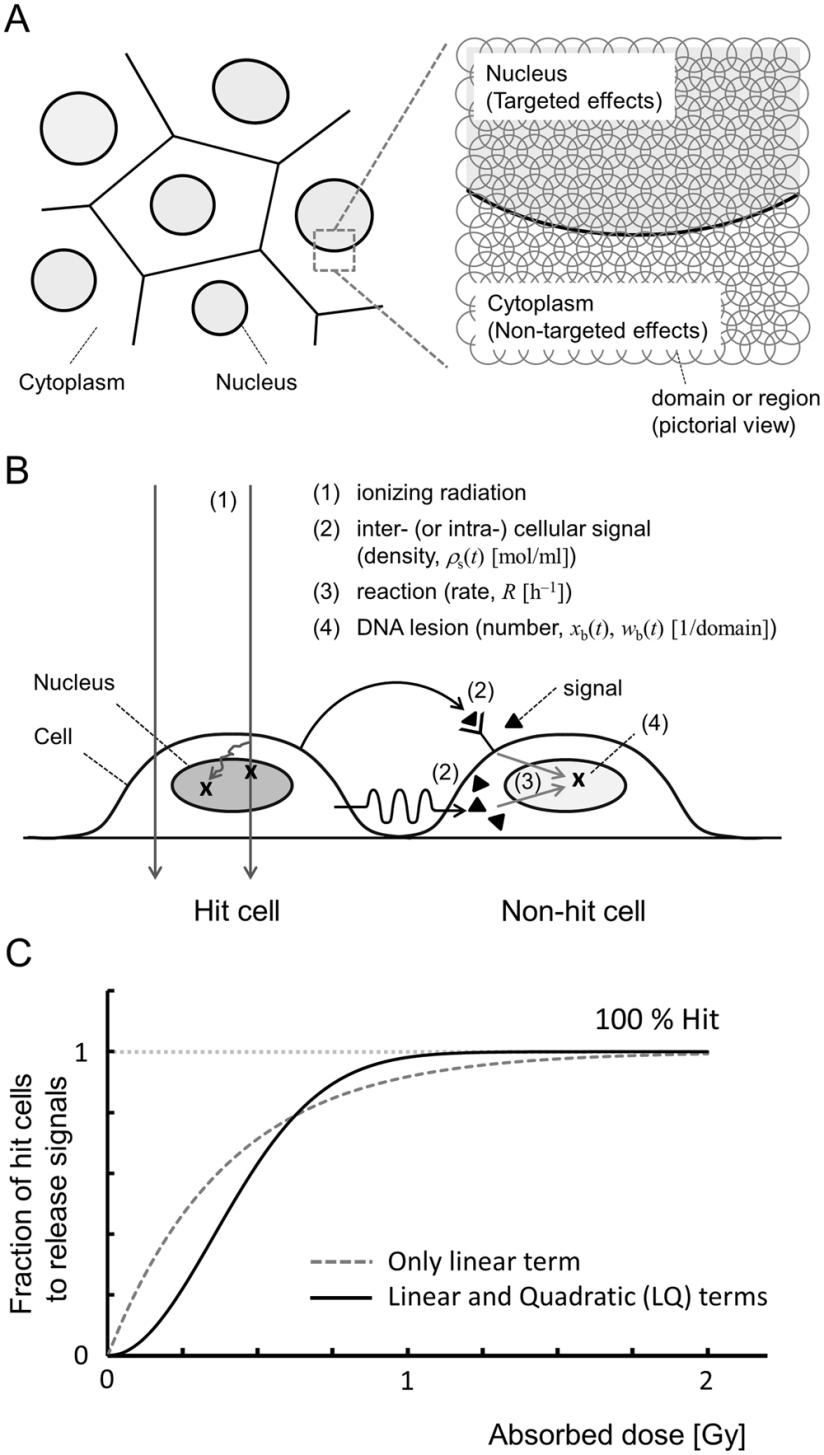
Conceptual illustration of the IMK model: (**A**) for micrometer-order targets (domains) in a cell population, (**B**) for processes that induce NTEs and (**C**) for the LQ relation to demonstrate the number of hits to targets to release signals in NTEs. The scenario of non-hit effects in Fig. 1(B) is as follows: (i) when a cell population is exposed to ionizing radiation, DNA lesions are generated along the track of ionizing radiation; (ii) hit cells emit initial signals which spread out and increase by cascade reactions as cell-killing signals (▲); (iii) the signals that reach to the neighboring cells (non-hit cells) induce potentially lethal lesions (PLLs) in proportion to the signal density; (iv) the PLLs may transform into lethal lesions (LLs) or be repaired.

In the MK model, a cell nucleus is divided into hundreds of micron-order territories (called domains) which are generally defined as spheres with 1–2 μm diameters (Fig. 1(A))^32^. The model considers microdosimetry by using specific energy *z* in Gy (dose per domain) or dose-mean lineal energy *y*_*D*_ in keV/μm. In this study, the site size is set to 1 μm diameter based on recent microdosimetric analysis combined with tissue equivalent proportional counter (TEPC)^44,45^. When a cell population is exposed to ionizing radiation, potentially lethal lesions (PLLs) may be induced along the radiation particle track passing through domains in cells. Every PLL has a possibility to be repaired. A PLL is assumed to undergo one of three transformations: (i) a PLL transforms into a LL via a first-order process at a constant rate *a* [h^−1^]; (ii) two PLLs interact with each other and transform into a LL via a second-order process at a constant rate *b*_d_ [h^−1^]; (iii) a PLL is repaired by a DNA repair function via a first-order process at constant rate *c* [h^−1^]. If the number of PLLs in a domain after acute irradiation is proportional to *z* (specific energy) and the DNA amount *g* in the domain^46^, the number of PLLs in the domain as a function of time after irradiation, *x*_d_(*t*), is described by

1 $$\begin{array}{ccc}\frac{{\rm{d}}}{{\rm{d}}t}{x}_{{\rm{d}}}(t) & = & -(a+c){x}_{{\rm{d}}}(t)-2{b}_{{\rm{d}}}{x}_{{\rm{d}}}{(t)}^{2},\\  & \cong  & -(a+c){x}_{{\rm{d}}}(t).\quad \because \,(a+c){x}_{{\rm{d}}}(t)\gg 2{b}_{{\rm{d}}}{x}_{{\rm{d}}}{(t)}^{2}\\ {x}_{{\rm{d}}}(t) & = & {k}_{{\rm{d}}}gz{e}^{-(a+c)t}.\end{array}$$

Here, we consider a single-dose continuous irradiation to a cell population with constant dose rate $\dot{D}$ [Gy/h] and dose-delivery time *T* [h]. According to a previous model^36,47^, by dividing the irradiation time *T* into *N* sections as *N* = *T/ΔT*, we can describe the discontinuous deposition of the energy into domains, where *ΔT* is a constant period of time. Let *z*_1_, *z*_2_, …, *z*_*N*_ and *g*_1_, *g*_2_, …, *g*_*N*_ be the specific energy and the DNA amount per domain, respectively, at every period, 0~*ΔT*, *ΔT*~2*ΔT*, …, (*N*−1)*ΔT*~*NΔT*. The number of PLLs per domain is given by,

2 $$\begin{array}{llll}{x}_{{\rm{d}}}(t) & = & {k}_{{\rm{d}}}{g}_{1}{z}_{1}{e}^{-(a+c)t} & [0\le t < \Delta T\,]\\ {x}_{{\rm{d}}}(t) & = & {\sum }_{n=1}^{2}{k}_{{\rm{d}}}{g}_{n}{z}_{n}{e}^{-(a+c)[t-(n-1)\Delta T]} & [\Delta T\le t < 2\Delta T]\\  & \vdots  &  & \\ {x}_{{\rm{d}}}(t) & = & {\sum }_{n=1}^{N-1}{k}_{{\rm{d}}}{g}_{n}{z}_{n}{e}^{-(a+c)[t-(n-1)\Delta T]} & [(N-2)\Delta T\le t < (N-1)\Delta T]\\ {x}_{{\rm{d}}}(t) & = & \,{\sum }_{n=1}^{N}{k}_{{\rm{d}}}{g}_{n}{z}_{n}{e}^{-(a+c)[t-(n+1)\Delta T]}. & [(N-1)\Delta T\le t]\end{array}$$

The number of LLs per domain, *w*_d_, can be expressed by the next equation,

3 $$\frac{{\rm{d}}}{{\rm{d}}t}{w}_{{\rm{d}}}=a{x}_{{\rm{d}}}(t)+{b}_{{\rm{d}}}{x}_{{\rm{d}}}{(t)}^{2}.$$

By solving Eq. 3 in the expanded form that includes the effect on every domain as an element of a nucleus and taking a limit of *N* to infinity to be equivalent to continuous irradiation (Supplementary information I), the surviving fraction for TEs after single-dose irradiation *S*_T_ can be expressed by

4a $$\begin{array}{rcl}{\langle w\rangle }_{{\rm{T}}} & = & p\langle {w}_{{\rm{d}}}\rangle \\  & = & \,({\alpha }_{0}+\frac{{y}_{D}}{{\rm{\rho }}{\rm{\pi }}{r}_{{\rm{d}}}^{2}}{\beta }_{0})\dot{D}T+\frac{2{\beta }_{0}}{{(a+c)}^{2}{T}^{2}}[(a\,+\,c)T+{e}^{-(a+c)T}-1]{(\dot{D}T)}^{2}\\  & = & ({\alpha }_{0}+\gamma {\beta }_{0})D+F{\beta }_{0}{D}^{2}\\  & = & -\mathrm{ln}\,{S}_{{\rm{T}}},\end{array}$$

where

4b $$F=\frac{2}{{(a+c)}^{2}{T}^{2}}[(a+c)T+{e}^{-(a+c)T}-1],$$

4c $$D\,=\,\dot{D}T,$$

4d $$\gamma =\frac{{y}_{D}}{\rho \pi {r}_{{\rm{d}}}^{2}},$$

and < *w* > _T_ is the average number of LLs per nucleus, *ρ* represents density (1.0 g/cm^3^) of the spherical domain with radius (0.5 μm), *y*_*D*_ is the dose-mean lineal energy (keV/μm), *F* corresponds to the Lea-Catcheside factor^48^, *p* is the number of domains per cell nucleus, *α*_0_ and *β*_0_ are cell-specific parameters defined by

4e $${\alpha }_{0}=\frac{a{k}_{{\rm{d}}}p\langle {g}_{0}\rangle }{(a+c)}\,$$

4f $${\beta }_{0}=\frac{{b}_{{\rm{d}}}{k}_{{\rm{d}}}2p\langle {g}_{0}^{2}\rangle }{2(a+c)}.$$

Here, *g*_0_ represents DNA amount per domain in kg. If the irradiation time *T* [h] is negligibly short in the special case of high-dose-rate irradiation, Eq. 4a can be approximated as the well-known linear-quadratic (LQ) model with the coefficients of *α* [Gy^−1^] and *β* [Gy^−2^] as,

5 $$\begin{array}{rcl}\mathop{\mathrm{lim}}\limits_{T\to 0}(-\mathrm{ln}\,{S}_{T}) & = & ({\alpha }_{0}+\gamma {\beta }_{0})D+{\beta }_{0}{D}^{2}\\  & = & \alpha D+\beta {D}^{2}.\end{array}$$

Equation 5 is an approximation formula of SF for a special case of acute irradiation. In this study, we used Eqs 2 and 3 to evaluate the DNA damage kinetics and Eq. 4 for describing the cell survival curve including the dose-rate for TEs (Supplementary information I).

## Model Overview- Intercellular Signalling

### Assumptions of non-targeted effects

In our NTE model, the scenario where the radiation-induced bystander effect (RIBE) leads to cell death assumes:

(i) Targets that emit the initial signals (stimulating substance) are regions somewhere in the cell of micron-order size as large as Mitochondria. The number of hits to the region follows a linear-quadratic function of specific energy. Note that the “hits” in this study do not mean the events such as ionizations and excitations but the target activation to release the signals after irradiation.
(ii) Initial signals originate and spread out in an area *r* μm away from the hit cells. Cell-killing signals are increased by signal cascade but are decreased by the decay of the signals and reaction to cells.
(iii) In the non-hit cells reacted by cell-killing signals, PLLs are induced in proportion to the signal concentration. According to the same constant rate of *a* [h^−1^] as the TEs^32^ and the repair rate in the non-hit cells as *c*_b_ [h^−1^], the signal-induced PLLs are transformed into LLs.
(iv) The number of LLs per nucleus in NTEs follows the Poisson distribution in the same manner as that of the TEs. Cell death is induced if the LLs remain in the cell nucleus.

The conceptual illustration of the scenario that cell-killing signals induce DNA lesions which lead to cell killing is summarized in Fig. 1(B).

### Target activation probability to emit cell-killing signals

In the present model, we assumed that the number of hits to the regions follows a linear-quadratic (LQ) shape as a function of specific energy. The LQ relation is mathematically useful for considering the hits by a single track and by two tracks (Fig. 1(C)). The signal entities are supposed to spread out when the regions are hit^10,11^. Region size is also assumed to be 1 μm in diameter in this study for the same reason as TEs. The number of hits (*N*_h_) per domain can be expressed by a linear-quadratic function as

6 $${N}_{{\rm{h}}}={A}_{{\rm{b}}}z+{B}_{{\rm{b}}}{z}^{2},$$

where *A*_b_ and *B*_b_ are the proportionality factors to *z* [Gy] and *z*^2^ [Gy^2^], respectively. Considering the probability density of *z*, the average number of hits per cell < *N*_h_ > is given by

7 $$\begin{array}{rcl}\langle {N}_{{\rm{h}}}\rangle  & = & {p}_{{\rm{b}}}{\int }_{0}^{\infty }({A}_{{\rm{b}}}z+{B}_{{\rm{b}}}{z}^{2})\,f\,(z)\,{\rm{dz}}\\  & = & ({\alpha }_{{\rm{b}}}+\gamma {\beta }_{{\rm{b}}})D+{\beta }_{{\rm{b}}}{D}^{2}\end{array},$$

where *p*_b_ is the number of regions for NTEs in a cell, and *α*_b_ = *p*_b_*A*_b_ and *β*_b_ = *p*_b_*B*_b_. The number of incident particles which traverse regions in each cell will follow the Poisson statistics because the probability of the traverse in a micro-order area is very low, especially in the case of low-dose exposure. So, the fraction of hit cells is expressed as

8 $$\begin{array}{rcl}{f}_{{\rm{h}}}(D) & = & 1-{e}^{-\langle {N}_{{\rm{h}}}\rangle }\\  & = & 1-\,{e}^{-({\alpha }_{{\rm{b}}}+\gamma {\beta }_{{\rm{b}}})D-{\beta }_{{\rm{b}}}{D}^{2}.}\end{array}$$

Equation 8 represents the dose-dependent probability of target activation, and this LQ formula considers the probability of hit to targets, which is an alternative function to the multi-target theory or threshold-like function^49^.

### Signal-induced DNA damage and cell survival

A series of cell responses from the signal concentration to DNA damage is summarized here. Referring to previous models of cell-killing signals, the cell-killing signal concentration *ρ*_s_(*r*, *t*) in an area *r* μm away from the hit cell (in diffusion area) at time (*t*) after irradiation [mol/ml] is expressed by

9 $${\rho }_{{\rm{s}}}(\underline{r},\,t)=\frac{{r}_{{\rm{s}}}\,{\mu }_{{\rm{s}}}\,{s}_{{\rm{d}}}\,(\underline{r})}{{\mu }_{{\rm{s}}}-(\lambda +R)}\{1-\,{e}^{-[{\mu }_{{\rm{s}}}-(\lambda +R)]t}\}{e}^{-(\lambda +R)t}$$

where *r*_s_ is the reactivation coefficient to produce the cell-killing signals; *λ* [h^−1^] is the constant rate for the cell-killing signal that decays exponentially (lifetime 1/*λ*); and *R* [h^−1^] is the constant rate for the cell-killing signals reacting with the nucleus of the non-hit cells.

Next, based on the new assumption (iii) about DNA damage kinetics, we deduced the temporal-dependence of signal-induced PLLs in NTEs. The PLLs are assumed to be induced in proportion to the amount of cell-killing signals, and the lesions have a potential to be repaired. The average number of the signal-induced PLLs, *x*_b_(*r*, *t*), per non-hit domain nearby hit domains follows the equation

10 $$\frac{{\rm{d}}}{{\rm{d}}t}{x}_{{\rm{b}}}\,(\mathop{r}\limits_{\_},\,t)={f}_{{\rm{b}}}(D){k}_{{\rm{b}}}R{\rho }_{{\rm{s}}}\,(\mathop{r}\limits_{\_},\,t)-(a+{c}_{{\rm{b}}}){x}_{{\rm{b}}}\,(\mathop{r}\limits_{\_},\,t),$$

where *k*_b_ is the number of the PLLs per domain caused by the signals [(mol/ml)^−1^], *a* is a constant rate to transform from PLL to LL [h^−1^] in the MK model^32^, *c*_b_ is a constant rate for repair in non-hit cells [h^−1^], and *f*_b_(*D*) denotes the fraction of non-hit cells in the cell population, i.e. *f*_b_(*D*) = 1−*f*_h_(*D*). By solving Eq. 10, we have

11 $${x}_{{\rm{b}}}\,(\underline{r},\,t)=\frac{{\rm{R}}\,{\mu }_{{\rm{s}}}\,{r}_{s}{k}_{{\rm{b}}}{s}_{{\rm{d}}}\,(\underline{r})\,{f}_{{\rm{b}}}(D)}{{\mu }_{{\rm{s}}}-({\rm{\lambda }}+{\rm{R}})}\{-\frac{1-\,{e}^{-[{\mu }_{{\rm{s}}}-(a+{c}_{{\rm{b}}})]t}}{{\mu }_{{\rm{s}}}-(a+{c}_{{\rm{b}}})}+\frac{1-{e}^{-[(\lambda +R)-(a+{c}_{{\rm{b}}})]t}}{(\lambda +R)-(a+{c}_{{\rm{b}}})}\}{e}^{-(a+{c}_{{\rm{b}}})t}.$$

Here, the average number of PLLs per domain is considered to be a spatially-dependent number. It should be noted that all the signals are released from hit cells that include hit regions. The average number of the PLLs per domain is given by

12 $$\,\begin{array}{ccc}\langle {x}_{{\rm{b}}}\rangle (t) & = & {\sum }_{P{f}_{{\rm{h}}}(D)}{x}_{{\rm{b}}}\,(\mathop{r}\limits_{\_},\,t)\\  & = & \frac{R\,{\mu }_{{\rm{s}}}{r}_{{\rm{s}}}{k}_{{\rm{b}}}{s}_{{\rm{P}}}{f}_{{\rm{b}}}(D)\,{f}_{{\rm{h}}}(D)}{{\mu }_{{\rm{s}}}-(\lambda +R)}\,\{-\frac{1-{e}^{-[{\mu }_{{\rm{s}}}-(a+{c}_{{\rm{b}}})]t}}{{\mu }_{{\rm{s}}}-(a+{c}_{{\rm{b}}})}+\frac{1-{e}^{-[(\lambda +R)-(a+{c}_{{\rm{b}}})]t}}{(\lambda +R)\,-(a+{c}_{{\rm{b}}})}\}{e}^{-(a+{c}_{{\rm{b}}})t}.\end{array}$$

where *s*_P_ represents the maximum amount of initial signals [mol/ml] and *P* is the total number of regions for the NTEs; therefore, if all regions are hit in the irradiated field, *s*_P_ is equal to <*s*_d_(*r*) > *P*. Thu*s*, Eq. 12 and the rate equation of the average number of LLs per domain can be linked as

13 $$\frac{{\rm{d}}}{{\rm{d}}t}{w}_{{\rm{b}}}=a\langle {x}_{{\rm{b}}}\rangle \,(t),$$

and we have

14 $${w}_{{\rm{b}}}=\frac{aR{r}_{{\rm{s}}}\,{k}_{{\rm{b}}}{s}_{{\rm{P}}}\,{f}_{{\rm{b}}}(D)\,{f}_{{\rm{h}}}(D)}{(\lambda +R)(a+{c}_{{\rm{b}}})}.$$

Let <*w*> _NT_ be the average number of LLs induced by the signals per cell nucleus, and we have

15 $$\,\begin{array}{rcl}{\langle w\rangle }_{{\rm{NT}}} & = & \sum _{p}{w}_{{\rm{b}}}\\  & = & \frac{aR{r}_{{\rm{s}}}{K}_{{\rm{b}}}{s}_{{\rm{P}}}}{(\lambda +R)(a+{c}_{{\rm{b}}})}[1-{e}^{-({\alpha }_{{\rm{b}}}+\gamma {\beta }_{{\rm{b}}})D-{\beta }_{{\rm{b}}}{D}^{2}}]{e}^{-({\alpha }_{{\rm{b}}}+\gamma {\beta }_{{\rm{b}}})D-{\beta }_{{\rm{b}}}{D}^{2}}\\  & = & \delta [1-{e}^{-({\alpha }_{{\rm{b}}}+\gamma {\beta }_{{\rm{b}}})D-{\beta }_{{\rm{b}}}{D}^{2}}]{e}^{-({\alpha }_{{\rm{b}}}+\gamma {\beta }_{{\rm{b}}})D-{\beta }_{{\rm{b}}}{D}^{2}}\end{array}$$

where

16 $$\delta =\frac{aR{r}_{{\rm{s}}}{K}_{{\rm{b}}}{s}_{{\rm{P}}}}{(\lambda +R)(a+{c}_{{\rm{b}}})}$$

and *p* is the number of domains per cell nucleus, *K*_b_ = *pk*_b_. Assuming that the number of LLs per nucleus follows the Poisson distribution, the expression of cell surviving fraction by the NTEs (*S*_NT_) is given from Eq. 15 as

17 $$\begin{array}{rcl}{\langle w\rangle }_{{\rm{NT}}} & = & \delta [1-{e}^{-({\alpha }_{{\rm{b}}}+\gamma {\beta }_{{\rm{b}}})D-{\beta }_{{\rm{b}}}{D}^{2}}]{e}^{-({\alpha }_{{\rm{b}}}+\gamma {\beta }_{{\rm{b}}})D-{\beta }_{{\rm{b}}}{D}^{2}}\\  &  & -\mathrm{ln}\,{S}_{{\rm{NT}}}.\end{array}$$

## Model Overview- Integrated Cell Survival and Modification for ICCM

It can be assumed that the possibility of interactions among PLLs in TEs and NTEs is very small at the domain level, so the lesions in TEs and NTEs can be treated as independent ones. To describe the surviving fraction of irradiated cells considering the TEs and NTEs, the number of LLs related with both effects (denoted as <*w* > _T_ and <*w* > _NT_, respectively) is written by

18 $$\langle w\rangle ={\langle w\rangle }_{{\rm{T}}}+{\langle w\rangle }_{{\rm{NT}}}.$$

Thus, the cell surviving fraction (*S*) is given by

19 $$S={S}_{{\rm{T}}}\times {S}_{{\rm{NT}}}.$$

Hereafter, we call this model the “integrated microdosimetric-kinetic (IMK) model” in this study.

The modelling of the NTEs takes account of the signal concentration and the number of DNA lesions as a function of time after irradiation. Taking advantage of this versatility, we next modified the IMK model to express the cell survival after exposure with irradiated cell culture medium (ICCM), namely medium transfer bystander effects (MTBEs).

As for the ICCM in a dish containing irradiated cells with dose *D*, the mean signal concentration in ICCM at time *t*_h_ [h] after irradiation can be expressed using Eq. 9 as

20 $$\begin{array}{ccc}\langle {\rho }_{{\rm{s}}}\,(\mathop{r}\limits_{\_},\,t)\,\rangle  & = & {\sum }_{P{f}_{{\rm{h}}}(D)}\{\frac{{r}_{{\rm{s}}}\,{\mu }_{{\rm{s}}}\langle {s}_{{\rm{d}}}\,(\mathop{r}\limits_{\_})\rangle }{{\mu }_{{\rm{s}}}-(\lambda +R)}[{e}^{-(\lambda +R){t}_{{\rm{h}}}}-{e}^{-{\mu }_{s}{t}_{{\rm{h}}}}]\}\\  & = & \frac{{r}_{{\rm{s}}}{\mu }_{{\rm{s}}}\,{s}_{{\rm{P}}}{f}_{{\rm{h}}}(D)}{{\mu }_{{\rm{s}}}-(\lambda +R)}[{e}^{-(\lambda +R){t}_{{\rm{h}}}}-{e}^{-{\mu }_{s}{t}_{{\rm{h}}}}].\end{array}$$

Then, after the transfer of the ICCM to the dish with the recipient cells, the signal concentration at time (*t*) is modified as

21 $$\langle {\rho }_{{\rm{s}}}(\underline{r},\,t)\rangle =\frac{{r}_{{\rm{s}}}\,{\mu }_{{\rm{s}}}\,{s}_{{\rm{P}}}\,{f}_{{\rm{h}}}(D)}{{\mu }_{{\rm{s}}}-(\lambda +R)}[{e}^{-(\lambda +R){t}_{{\rm{h}}}}-{e}^{-{\mu }_{s}{t}_{{\rm{h}}}}]{e}^{-(\lambda +R)t}.$$

Thus, the average number of the PLLs per recipient domain is deduced to

22 $$\frac{{\rm{d}}}{{\rm{d}}t}\langle {x}_{{\rm{b}}}\rangle \,(t)={k}_{{\rm{b}}}R{\rho }_{{\rm{s}}}\,(t)-(a+{c}_{{\rm{b}}})\langle x{\rm{b}}\rangle \,(t),\quad \langle {x}_{{\rm{b}}}\rangle \,(0)=0,$$

then

23 $$\langle {x}_{{\rm{b}}}\rangle \,(t)=\frac{R{\mu }_{{\rm{s}}}\,{r}_{{\rm{s}}}\,{k}_{{\rm{b}}}{s}_{{\rm{P}}}\,{f}_{{\rm{h}}}(D)[{e}^{-(\lambda +R){t}_{{\rm{h}}}}-{e}^{-{\mu }_{s}{t}_{{\rm{h}}}}]}{{\mu }_{{\rm{s}}}-(\lambda +R)}\cdot \frac{{e}^{-(a+{c}_{{\rm{b}}})t}-{e}^{-(\lambda +R)t}}{(\lambda +R)-(a+{c}_{{\rm{b}}})},$$

and we have the formula by summing up over all domains (*p*) as

24 $$\begin{array}{rcl}{\langle w\rangle }_{{\rm{NT}}} & = & {\sum }_{p}{w}_{{\rm{b}}}\\  & = & \frac{aR{\mu }_{{\rm{s}}}\,{r}_{{\rm{s}}}\,{K}_{{\rm{b}}}{s}_{{\rm{P}}}\,{f}_{{\rm{h}}}(D)\,}{(\lambda +R)(a+{c}_{{\rm{b}}})}\cdot \frac{[{e}^{-(\lambda +R){t}_{{\rm{h}}}}-\,{e}^{-{\mu }_{{\rm{s}}}{t}_{{\rm{h}}}}]}{{\mu }_{{\rm{s}}}-(\lambda +R)}.\end{array}$$

Finally, we have a relational expression of the cell surviving fraction with the recipient cell population as

25 $$-\,\mathrm{ln}\,{S}_{{\rm{NT}}}={\delta }_{{\rm{mt}}}[1-\,{e}^{-({\alpha }_{{\rm{b}}}+\gamma {\beta }_{{\rm{b}}})D-{\beta }_{{\rm{b}}}{D}^{2}}].$$

where

26 $${\delta }_{{\rm{mt}}}=\frac{aR{\mu }_{{\rm{s}}}\,{r}_{{\rm{s}}}\,{K}_{{\rm{b}}}{s}_{{\rm{P}}}\,}{(\lambda +R)(a+{c}_{{\rm{b}}})}\cdot \frac{[{e}^{-(\lambda +R){t}_{{\rm{h}}}}-\,{e}^{-{\mu }_{{\rm{s}}}{t}_{{\rm{h}}}}]}{{\mu }_{{\rm{s}}}-(\lambda +R)}.$$

This equation represents the cell survival after exposure with ICCM, by which the probability of hit to regions in NTEs can be analyzed to interpret the underlying mechanism of signal emission quantitatively through the comparison with experimental MTBE data.

## Application of IMK Model to Experimental Data

To determine the cell-specific parameters in the IMK model, the formulae were fitted to the data by using the maximum-likelihood procedure with a Monte Carlo technique (Supplementary information II).

### Fitting to intercellular signalling and induced DNA damage data

Responses to intercellular signaling are characterized by *r*_s_*s*_d_(*r*), *μ*_s_ and (*λ* + *R*) in Eq. 9. We fit Eq. 9 to the relative signal concentration data reported by Lyng *et al*. (2002) for calcium as the first messenger of the signals^50^ and by Han *et al*. (2007) for NO as the final messenger^51^. We then obtained the parameters, *μ*_s_ and (*λ* + *R*), for calcium and NO.

DNA damage induction and its repair kinetics are characterized by *a*, *b*_d_, *c, γ* and *k*_d_ < *g* > for TEs and *c*_b_, *Rr*_s_*k*_b_*s*_p_, μ_s_, (*λ* + *R*), *α*_b_, *β*_b_ and *γ* for NTEs. The damage kinetics at the domain level in TEs and NTEs can be expressed by Eqs 2, 3, 10 and 13. By using these equations for the cell nucleus composed of *p* domains, we compared the average number of DSB per nucleus estimated by the model with experimental DSB data in primary normal human fibroblasts from the lung, MRC-5^24^. The response parameters of DNA damage link to cell survival parameters of *α*_0_ and *β*_0_, thus these can be determined backward from the parameters featuring cell survival according to the following procedure:

(i) The *γ* value, as a representative of microdosimetric quantity for both effects, for 200 kVp X-rays was taken from a previous report^52^.
(ii) (The values of (*a* + *c*) and *k*_d_ < *g* > were obtained from two reports on mammalian cell lines^32,35^. Then, by using Eqs 4e,f, *a*, *b*_d_, and *c* values were deduced backward from the survival-specific parameters (*α*_0_ = 0.358 [Gy^−1^], *β*_0_ = 0.0618 [Gy^−1^]) in a normal human fibroblast cell line^53^. Note that we used the values, *p* = 9.55 × 10^2^ calculated from a report on cell size^54^ and Φ = 1.04 for the plateau phase from the cell-cycle data^55^.
(iii) After fixation of the parameters in TEs (*a*, *b*_d_, *c*, *γ*, *k*_d_ < *g >*), we determined the cell specific parameters *c*_b_, *Rr*_s_*k*_b_*s*_p_, *α*_b_, *β*_b_ by using μ_s_, (*λ* + *R*) in the specific case of calcium in NTEs by fitting the formulae (Eqs 2, 3, 10 and 13) to the DSB data^24^.

### Fitting to dose-response curve and MTBE data

The dose-response curve of cell survival (hereafter, the cell survival curve) is characterized by 7 parameters, *α*_0_, *β*_0_ and (*a* + *c*) in TEs, *α*_b_, *β*_b_ and *δ* in NTEs, and *γ* in both effects. We applied Eqs 4, 17 and 19 to the cell survival data for V79–379A^56–61^ and T-47D cell lines. The *γ* value was taken from previous reports^35,52^, and the residual cell-specific parameters were determined all at once. By using these parameters, we illustrated cell survival curves in comparison with the experimental survival data for the V79-379A^56–61^ and T-47D cell lines^62–65^.

The other SF data of HPV-G cells after exposure with ICCM^11,66–69^ and that of CHO-K1 cells after irradiation of broad beam X-rays^70^ were also fitted by the IMK model, and all parameters in the model were determined simultaneously. Whilst the data in MTBEs was used to evaluate the linear-quadratic relation of target activation to release signals, a set of SF data in CHO-K1 cells was used to investigate the relation between the low-dose HRS and the inactivation of the repair in non-hit cells.

### Fit quality

To check the fit quality of the IMK model to the experimental data of signals, DNA damage kinetics and cell survival, we calculated the *R*^2^ value given by

27 $${R}^{2}=1-\frac{{\sum }_{i={\mathfrak{1}}}^{n}{(Ex{p}_{i}-Mo{d}_{i})}^{2}\,/(n-m-1)}{{\sum }_{i={\mathfrak{1}}}^{n}{(Ex{p}_{i}- < Exp > )}^{2}/(n-1)},$$

where *Exp* represents the experimental value and *Mod* is for the calculated value by the IMK model, *n* is the number of data, *m* is the number of parameters in the model, and *n*−*m*−1 represents the degree of freedom. In addition to the *R*^2^ value, for evaluating model selection in NTEs we calculated the chi-square value and Akaike’s information criterion (AIC)^71^ for the data in MTBEs. The chi-square and AIC values are defined by

28a $${\chi }^{2}={\sum }_{i={\mathfrak{1}}}^{n}\frac{{({S}_{\exp i}-{S}_{{\rm{m}}{\rm{o}}{\rm{d}}{\rm{e}}{\rm{l}}i})}^{2}}{{\rm{\Delta }}{{S}_{\exp i}}^{2}},$$

28b $${\rm{A}}{\rm{I}}{\rm{C}}=n\,{\rm{l}}{\rm{n}}[\frac{{{\sum }_{i={\mathfrak{1}}}^{n}({S}_{\exp i}{\mathfrak{-}}{S}_{{\rm{m}}{\rm{o}}{\rm{d}}{\rm{e}}{\rm{l}}i})}^{2}}{n}]+2m,$$

where *S*_exp_ is the experimental surviving fraction, *S*_model_ is the surviving fraction calculated by the model and *ΔS*_exp_ is the experimental uncertainty.

## Results

### Temporary-dependence on response to signals and DSBs

**Figure 2:**
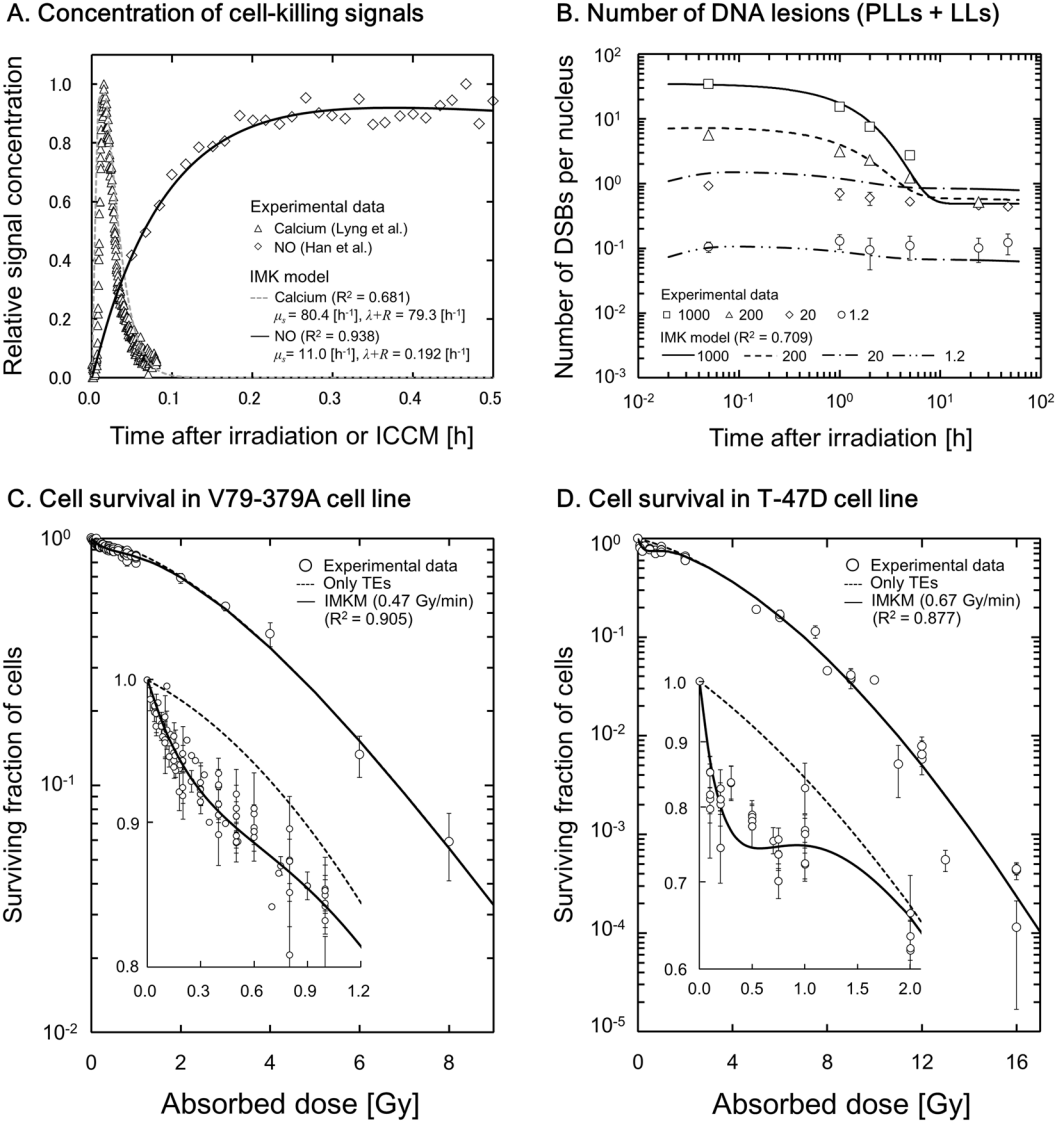
(**A**) and (**B**) show the comparison of the IMK model curve with experimental data for temporary dependence of cell-killing signals and of DNA double-strand breaks (DNA-DSBs) per nucleus, respectively. In Fig. 2(A), the specific signals, calcium as the first messenger^50^ and NO as the final messenger^51^, were adopted. Response parameters to DNA-DSBs are listed in Table 1. In Fig. 2(B), the number represents the prescribed dose to cells in mGy. Under the assumption that the repair in non-hit cells is inactivated, the IMK model reproduced the time dependency of DNA-DSBs in good agreement with experimental data^24^. (**C**) and (**D**) represent the fitting results of the IMK model to the experimental SF data: (**C**) for the V79-379A cells and (**D**) for the T-47D cell line, respectively. The symbols represent the experimental SF data reported by Marples *et al*.^56–61^ and Edin *et al*.^62–65^. The black solid line and dotted line represent the curve described by the IMK model with or without NTEs, respectively. The parameters in the model are summarized in Table 2. The IC in Fig. 2 means intercellular communication.

Figure 2(A) shows the changes in cell-killing signal concentration of calcium and NO after irradiation or ICCM, in which the model well reproduces experiments^50,51^ with good *R*^2^ values. The parameters for cell-killing signal, *μ*_s_ and (*λ* + *R*), for calcium are 80.4 [h^−1^] and 79.3 [h^−1^] and those for NO are 11.0 [h^−1^] and 0.192 [h^−1^], respectively. According to Hu *et al*.^23^, the number of DSBs in irradiated and in non-irradiated cells reaches its peak at 30 min after radiation. Since the damage induction in the NTEs may have occurred at an earlier time after irradiation, we next tried to reproduce the kinetics of the number of DSBs per nucleus induced by NTEs, assuming that the first messenger of calcium induces the damage. Figure 2(B) shows fitting results of the IMK model to the DSB kinetics data in MRC-5 (human normal fibroblast cell line^24^), in which the DSB kinetics curve in the IMK model was described using the parameters summarized in Table 1. The black lines and symbols in Fig. 2(B) represent the curves by the IMK model (Eqs 2, 3, 12 and 13) and experimental data^24^, respectively. The number in Fig. 2(B) represents the prescribed dose in mGy. The curves by the IMK model in consideration of inactivation of the repair in non-hit cells agree well with the experimental data.

**Table 1:** Parameters associated with signal concentration (calcium) and the number of DNA lesions in human fibroblast cells.

**Concentration of intercellular signaling**		
*μ*_s_	80.4	[h^−1^]
*λ* + *R*	79.3	[h^−1^]
**DNA damage kinetics (in case of calcium)**		
*a*	9.37 × 10^−3^	[h^−1^]^*^
*b*_d_	1.15 × 10^−1^	[h^−1^]^*^
*a* + *c*	7.04 × 10^−1^	[h^−1^]^**^
*p*	9.55 × 10^2^	[per nucleus]***
*k*_d_ < *g* >	2.83 × 10^−2^	[Gy^−1^]
Φ	1.04	(dimensionless)
*a* + *c*_b_	1.09 × 10^−2^	[h^−1^]
*R r*_s_ *k*_b_ *s*_P_	4.61 × 10^−1^	[h^−1^]
**Number of hit probability for NTEs**		
*α*_b_	5.38	[Gy^−1^]
*β*_b_	5.41	[Gy^−2^]
*γ*	0.923	[Gy] (200 kVp X-rays)

^*^a and *b*_d_ were deduced by using Eq. 4 with the parameters (*α*_0_, *β*_0_, *k*_d_*p* < *g* >). **(*a* + *c*) value in mammali*a*n *c*ells was taken from ref.^35^. ***p was calculated from the sizes of domain (*ϕ* 1.0 μm) and volume of nucleus (500 μm^3^) as in ref.^54^.

### Cell survival curve described by the present model

Table 2 summarizes the parameters associated with cell survival in the IMK model. Figure 2(C,D) show the fitting results of the IMK model for V79-379A cell line and for T-47D cell line, respectively. In these figures, the solid line is the curve given by the IMK model (TEs and NTEs) and the dotted line is by the model considering only TEs. The symbols represent the experimental SF data^56–65^. Taking account of NTEs in the IMK model, the low-dose HRS for both cell lines was reproduced well by the use of Eqs 4, 17 and 19. As shown in Fig. 2, the IMK model can precisely reproduce the low-dose HRS in terms of the *R*^2^ value (Supplementary information III).

**Table 2:** Parameters in the IMK model determined by maximum likelihood method.

Effect type	Parameter	Cell line			
		V79-379A	T-47D	HPV-G	E48
Targeted	*α*_0_ [Gy^−1^]	1.60 × 10^−2^	1.29 × 10^−1^	—	—
	*β*_0_ [Gy^−2^]	6.00 × 10^−1^	2.90 × 10^−2^	—	—
	(*a* + *c*) [h^−1^]	6.29	1.60	—	—
Common	*γ* [Gy]*	9.24 × 10^−1^	4.80 × 10^−1^	4.80 × 10^−1^	4.80 × 10^−1^
Non-Targeted	*α*_b_ [Gy^−1^]	1.46	1.80	3.09 × 10^1^	<1.00 × 10^−3^
	*β*_b_ [Gy^−2^]	3.96 × 10^−2^	3.00 × 10^−2^	2.38 × 10^−1^	1.72 × 10^−1^
	*δ* (*δ*_*m*_)	2.57 × 10^−1^	9.02 × 10^−1^	5.29 × 10^−1^	5.79 × 10^−1^

^*^The *γ* -values for 250 kVp X-rays and ^60^Co *γ*-rays were taken from refs^35,44^.

**Figure 3:**
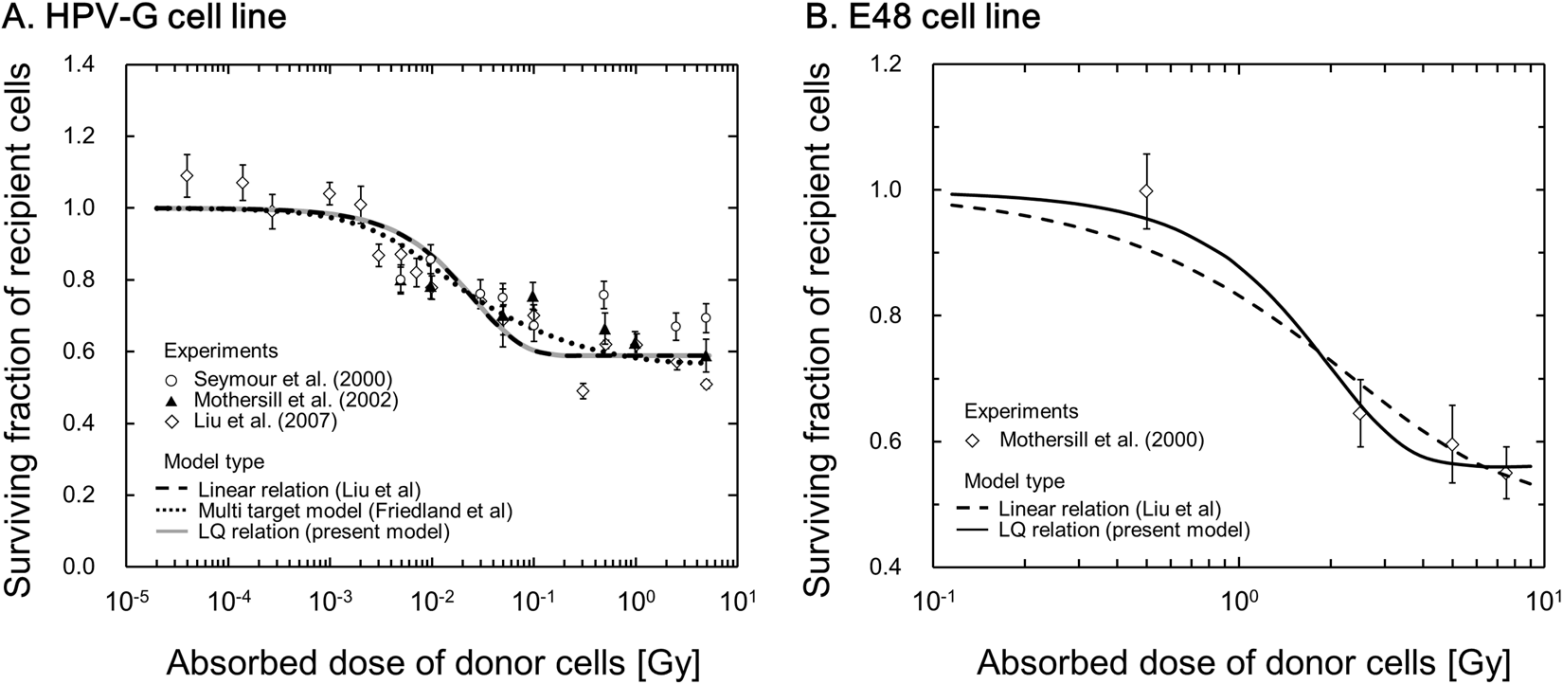
Comparison between the modified IMK model (Eq. 25) and experimental SF data in MTBEs^11,66–69^. The figures show the relation between the surviving fraction of recipient cells affected by the ICCM and absorbed dose in the irradiated (donor) cell population: (**A**) for HPV-G cell line and (**B**) for E48 cell line, respectively. It is noted that the quadratic term in Eq. 7 significantly contributes to the reproduction of cell survival in Fig. 3(B).

To evaluate the hypothesized mechanism of hits to targets in NTEs, the IMK model for MTBEs was further applied to the MTBE data^11,66–69^, where the parameters (in Eq. 25) in Table 2 were used as well. Figure 3(A,B) show the fitting curves of the IMK model to surviving fractions (SFs) for MTBEs in comparison with the experimental data for HPV-G and E48 cell lines. In Fig. 3(A,B), the horizontal axis is the absorbed dose in the irradiated cell population. The agreement between the resultant curves and the experimental data is also fair. The fitting results shown in Figs 2 and 3 suggest that the IMK model can describe not only the low-dose SF after irradiation with a broad beam but also the reduction of SF by intercellular signaling in MTBEs.

### Relation between low-dose HRS and repair in non-hit cells

**Figure 4:**
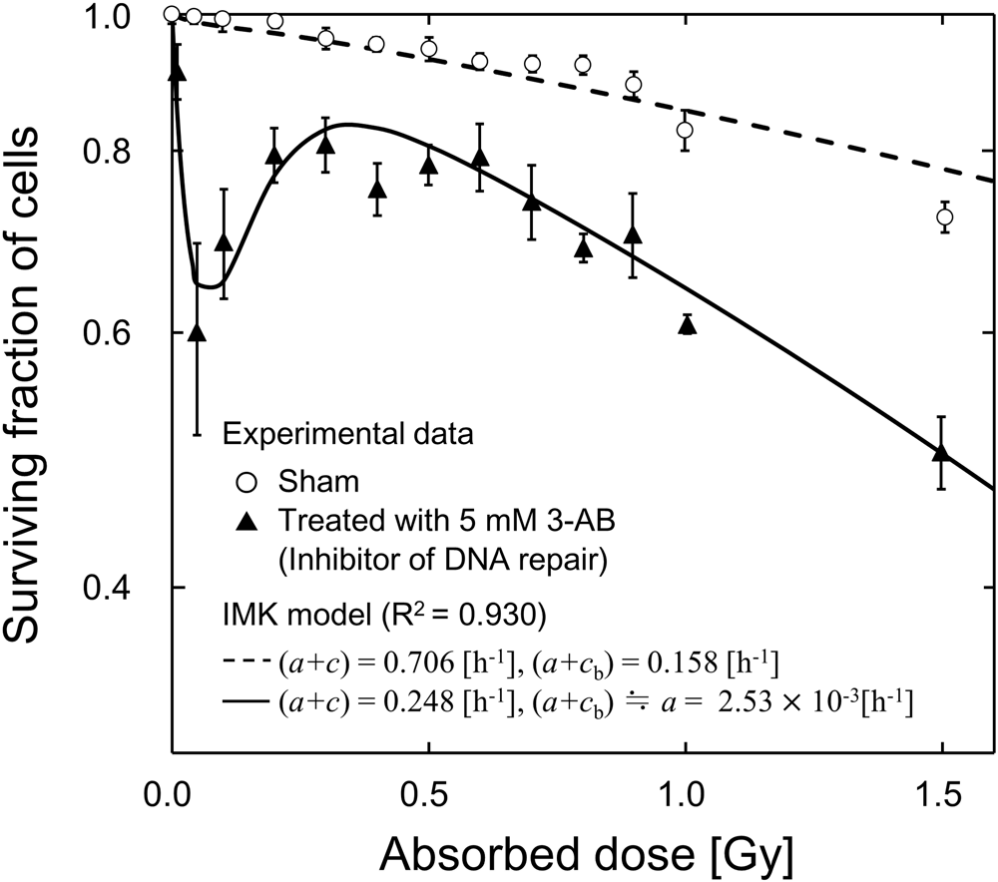
Comparison between the surviving fraction in our model and the experimental data for non-treated CHO-K1 cells and repair-inhibited cells^70^. The sets of response parameters in the cells are (*α*_0_, *β*_0_) = (1.15 × 10^−1^, 2.20 × 10^−2^) for TEs, (*α*_b_, *β*_b_, *δ*) = (9.28, 1.21, 2.79 × 10^−2^) for NTEs, and the common microdosimetric quantity *γ* = 0.924. To reproduce the experimental results by using the IMK model, the parameters (*α*_0_ ∝ *a*/(*a* + *c*), *β*_0_ ∝ *b*_d_/2(*a* + *c*) and *δ* ∝*a*/(*a* + *c*_b_)) of non-treated CHO cells to the repair-inhibited cells are chosen to be 3.52 × 10^−1^ for the TEs (*α*_0_, *β*_0_) and 1.60 × 10^−2^ for the NTEs (*δ*).

In the present model, the DNA repair function in non-hit cells is newly introduced as an inactivation factor. The assumption that the DNA repair in non-hit cells is inactivated was checked by the fitting results of DNA lesions as shown in Fig. 2(B). Next, focussing on inactivated repair, the IMK model was fitted simultaneously to the experimental data in both sham CHO-K1 cells and cells treated with an inhibitor of DNA repair^70^. Figure 4 shows the fitting results of the IMK model to experimental data. The sets of response parameters in the cells are (*α*_0_, *β*_0_) = (1.15 × 10^−1^, 2.20 × 10^−2^) for TEs, (*α*_b_, *β*_b_, *δ*) = (9.28, 1.21, 2.79 × 10^−2^) for NTEs, while the common microdosimetric quantity *γ* = 0.924 is chosen for 240 kVp X-rays (as it is close to an energy of 250 kVp X-rays^35^). The DNA repair functions for hit and non-hit cells are characterized by *a*/(*a* + *c*) (∝ *α*_0_) and *b*_d_/2(*a* + *c*) (∝ *β*_0_) in Eq. 4e,f for TEs, and *a*/(*a* + *c*_b_) (∝ *δ*) in Eq. 16 for NTEs, respectively. Here, we assumed that *a* and *b*_d_ are cell-specific parameters in the IMK model. To reproduce the experimental SF by using the IMK model, the parameters (*α*_0_ ∝ *a*/(*a* + *c*), *β*_0_ ∝ *b*_d_/2(*a* + *c*) and *δ* ∝*a*/(*a* + *c*_b_)) of non-treated CHO cells to the repair-inhibited cells were determined to be 3.52 × 10^−1^ for TEs (*α*_0_ and *β*_0_) and 1.60 × 10^−2^ for NTEs (*δ*). In the course of the model analysis about DNA repair efficiency of non-hit cells *c*_b_, it is suggested that the repair in non-hit cells is almost inactivated. For this reason, we estimated the repair rate of inactivation (*c*_b_) from parameters, *α*_0_ = 1.15 × 10^−1^ [Gy^−1^], *k*_d_*p* < *g >* = 32.1 [Gy^−1^]^52^ and (*a* + *c*) = 0.706 [h^−1^]^35^, to be *c*_b_ = 0.155 [h^−1^] with *a* = 2.52 × 10^−3^ [h^−1^]. The result in Fig. 4 and the estimated value of *c*_b_ suggest that the repair function in non-hit cells can be regarded as a key to reproduce the low-dose HRS in repair-inhibited CHO cells.

## Discussion

### Hit probability of emitting cell-killing signals in NTEs

The number of hits to targets to release signals in NTEs and the signal amount have been thought to be related with the mechanism as a function of absorbed dose in Gy or specific energy^11,12^. In the present model, we defined a formula for the mechanism as a LQ relation. To evaluate this definition, we further compared the IMK model with the previous model (linear relation) reported by Liu *et al*.^11^ in SF data after exposure with ICCM. The sets of parameters by Liu *et al*. (*z*_F_, *ω*) for MTBEs in HPV-G and E48 cell lines are (3.18 × 10^−2^, 5.29 × 10^−1^) and (3.10, 6.67 × 10^−1^), respectively. Whilst the quadratic term is close to zero in the HPV-G cell line (Table 2 and Fig. 3(A)), the linear term is close to zero (<0.001) in the E48 cell line (Table 2 and Fig. 3(B)). Table 3 shows that the chi-square value deduced by the LQ relation (with parameter number, *m* = 4) has a tendency to be smaller than the value by linear relation (*m* = 2).

**Table 3:** Comparison of fitting properties among the models for hit mechanisms in NTEs.

Type of cell line	Model type	Parameter number	Statistical index		
		*m*	*R* ^2^	*χ* ^2^	AIC
HPV-G	Liu *et al*.^11^	2	0.708	222	−158
	Friedland *et al*.^12^	11	0.704	153	−151
	Modified IMK model	4	0.687	222	−154
E48	Liu *et al*.^11^	2	0.948	3.22	−26.7
	Modified IMK model	4	0.982	0.934	−30.7

In addition, we fitted the previous model by Friedland *et al*.^12^ to MTBE data, in which the response parameter is *a* = 5.65 × 10^−1^ and the characteristic dose for signal emission *D*c values lies in between 10 and 1000 mGy (40% of cells with *D*c = 10 mGy, 20% with 30 mGy, 20% with 100 mGy, 10% with 300 mGy and 10% with 1000 mGy). Although the model (including a lot of parameters) by Friedland *et al*. makes the chi-square value the smallest, the AIC value as the index of model selection becomes larger.

It is generally accepted that the LQ relation in hit number depends on the number of radiation particles in the LQ model^72^. Collectively, the number of hits to targets in NTEs may hold similarly to that in the previous TEs.

### Model parameters and mechanisms of signal-induced cell-killing

The parameters, *α*_b_ and *β*_b_, were newly defined in this study. The parameters (*α*_b_ + *γβ*_b_) and *β*_b_ in Eq. 7 represent the proportionality factors to *D* and *D*^2^ in Gy^−1^ and Gy^−2^, and the reciprocals of (*α*_b_ + *γβ*_b_) and *β*_b_^1/2^ denote the doses to induce a signal-release hit with single particle track and the hit with a pairwise combination of two tracks, respectively. From Table 2, the values of 1/(*α*_b_ + *γβ*_b_) are given to be 0.68 Gy for V79-379A cells and 0.56 Gy for T-47D cells, while the values of 1/*β*_b_^1/2^ are 5.03 Gy for V79-379A cells and 5.77 Gy for T-47D cells. As shown in Table 1 (for the human fibroblast cell line) and Table 2 (for the V79-379A and T-47D cell lines), the values of *α*_b_ and *β*_b_ vary depending on the cell line. This suggests that the parameters in NTEs are cell-specific.

**Figure 5:**
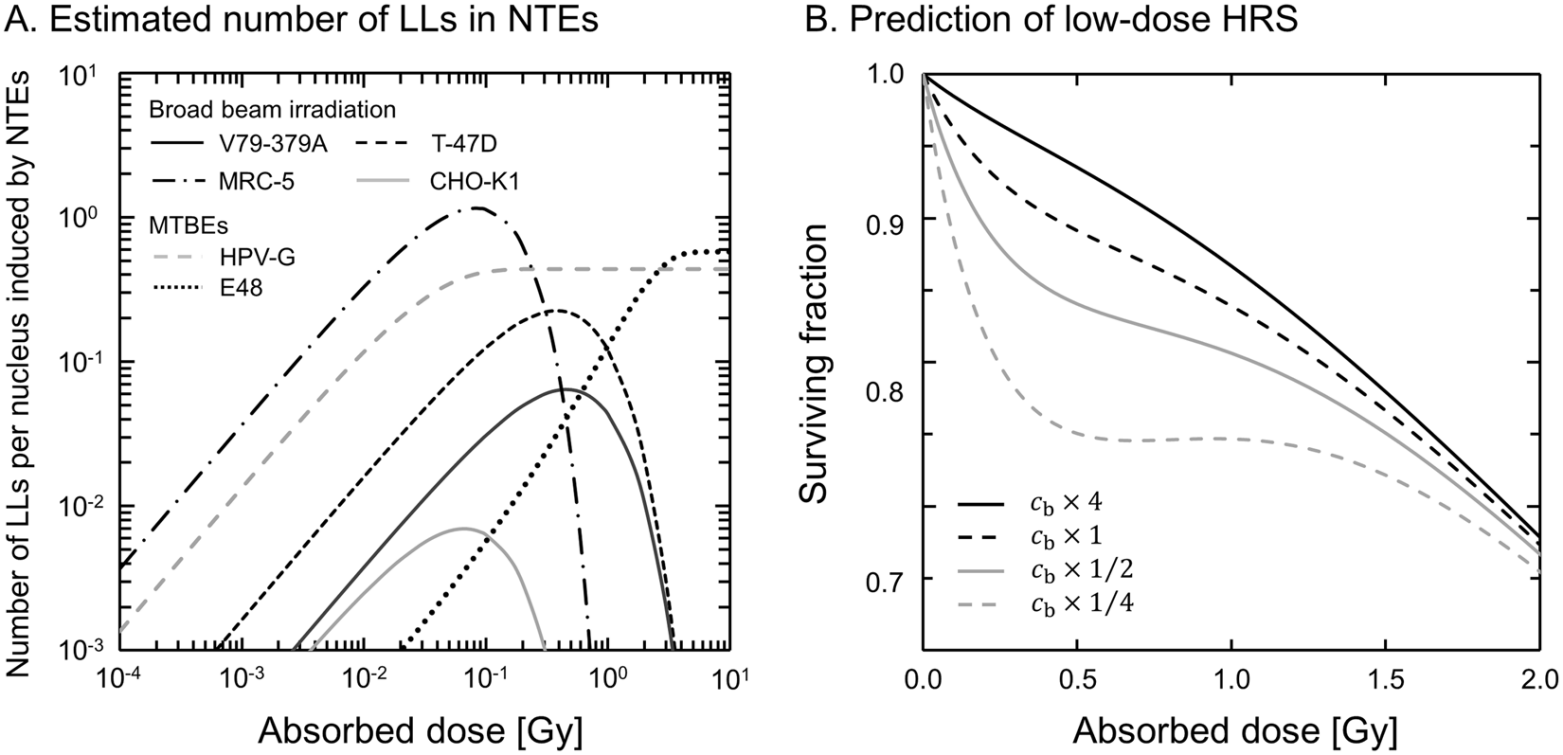
The IMK model analyses with the number of LLs in NTEs and DNA repair function in non-irradiated cells: (**A**) for the mean number of LLs per nucleus induced by NTEs in various cell lines, (**B**) for the surviving fraction with a variety of DNA repair factors. In Fig. 5(A), we used the IMK parameters deduced in this study to estimate LL number per nucleus. In Fig. 5(B), the cell survival curves for V79-379A cells were estimated with repair function rates, *c*_b_ × 4, *c*_b_ × 1, *c*_b_ × 1/2 and *c*_b_ × 1/4 under the condition of *a* = 8.12 × 10^−3^ [h^−1^].

By using the IMK parameters listed in Tables 1 and 2, we can estimate the degree of the dose-dependent NTEs based on the linear-quadratic theory. Figure 5(A) exemplifies the estimated number of LLs per nucleus for the NTEs. In Fig. 5(A), the maximum numbers of LLs per nucleus for V79-379A and T-47D cells are 0.064 and 0.23, respectively. The maximum number of LLs is characterized by the *δ* value in the present model. This value may also depend on the cell type. In the MRC-5 cell line, the repair kinetics of DNA damage after acute irradiation (Fig. 2B) and the number of LLs per nucleus were described based on calcium-induced DNA damage. Fernandez-Palomo *et al*. measured dose-dependence of calcium through the cellular membrane, indicating a possible link between low-dose HRS and bystander effects^73^. The report by them supports our model approach to estimate the DSB number per nucleus in NTEs and cell kill in NTEs.

### Involvement of the repair in low-dose HRS

Some previous investigations indicate that the defect of DNA repair in non-irradiated cells is related to the bystander effect^27,28^. According to the report by Rothkamm *et al*.^26^, the threshold value to activate DNA repair is a small dose such as 1.2 mGy, where one electron track may traverse a cell nucleus at most. Thus, it is reasonable to suppose that the DNA repair function in non-irradiated cells is inactivated. From the viewpoint of the repair capability in a cell population, it has been interpreted as that the increased radioresistance (IRR) is associated with overcoming low-dose HRS^30^. In contrast, we evaluated the influence of disorder of DNA repair function in non-hit cells on the NTE-related DSBs induction as shown in Fig. 2(B) and also cell death in Fig. 4.

Figure 5(B) illustrates the estimated curves for V79-379A cells in the IMK model with DNA repair *c*_b_ by factors of 4, 1, 1/2 and 1/4, in which the constant rate of *a* was determined to be 8.12 × 10^−3^ [h^−1^] from *k*_d_*p* < *g* > = 30 [Gy^−1^]^74^ and *α*_0_ = 3.89 × 10^−2^ [Gy^−1^], and *c*_b_ was estimated to be 0.155 [h^−1^] from the results in Fig. 4. As is shown in Fig. 5(B), the low-dose HRS is enhanced by lowering the repair factor. This suggests that the inactivation of DNA repair in non-hit cells tends to enhance cell-killing (or to decrease radioresistance) after a low-dose irradiation. As to the increase of repair by virtue of the repair function, evidence have been reported^75,76^.

In this study, through the analyses using the developed IMK model, we have demonstrated NTEs by the combination of a variety of processes: signal transfer from hit cells, kinetics of DSBs to enhance cell killing and disorder of the DNA repair function, to show characteristics of the radiosensitivity of cells in conformity with experimental evidences.

## Conclusion

In this study, an integrated microdosimetric-kinetic (IMK) model taking account of NTEs was applied to demonstrate the experimental data of the cell-killing signals, number of DSBs per nucleus and cell survival. From the comparison of the results by the model with experimental data, it was shown: (i) a LQ relation to express the hit probability for emitting signals is suitable to describe cell killing in NTEs, (ii) low-dose hyper-radiosensitivity (HRS) is attributed to the combination of the induction of DSBs by the signals and low DNA repair efficiency in non-hit cells, and (iii) the low-dose HRS is enhanced more as the DNA repair efficiency in non-hit cells is lower.

The IMK model provides quantitative formulae that enable us to analyze both TEs and NTEs based on cell-killing signals, DNA damage and DNA repair. We found that the inactivation of DNA repair in non-hit cells is dominant in HRS for cell survival.

## Electronic supplementary material

Supplementary information
