# Supplementary material for: Integrated Modelling of Cell Responses after Irradiation for DNA-Targeted Effects and Non-Targeted Effects
Source: Sci Rep. 2018 Mar 19;8:4849. doi: 10.1038/s41598-018-23202-y (PMC5859303; doi:10.1038/s41598-018-23202-y)
Supplement: Supplementary file 1 — Supplementary information [file 41598_2018_23202_MOESM1_ESM.pdf]

Integrated Modelling of Cell Responses after Irradiation for DNA-Targeted Effects and Non-Targeted Effects

Yusuke Matsuya, Kohei Sasaki, Yuji Yoshii, Go Okuyama and Hiroyuki Date

SPPLEMENTARY INFORMATION

I. *Deduction of cell survival formula after irradiation with a constant dose rate*

The derivation of the model for TEs is described below. Equation 2 is substituted into Eq. 3 for each period of time (ΔT). The accumulated number of LLs per domain is given by

$$w_\mathrm{d} = A\sum_{n=1}^{N}\left(g_n z_n\right) + B\sum_{n=1}^{N}\left(g_n{}^2 z_n{}^2\right) + 2B\left\{\sum_{n=1}^{N-1}\sum_{m=n+1}^{N}\left[e^{-(m-n)(a+c)\Delta T}\right]g_n g_m z_n z_m\right\}, \quad \text{(SI-1)}$$

where

$$A = \frac{ak_\mathrm{d}}{(a+c)} \text{ and } B = \frac{b_\mathrm{d}k_\mathrm{d}{}^2}{2(a+c)}. \quad \text{(SI-2)}$$

Let $<w_\mathrm{d}>$ the average number of w_d per domain, thus average number of LLs per cell nucleus $<w>_\mathrm{T}$ is expressed as

$$\begin{aligned}\langle w\rangle_\mathrm{T} &= \sum_{i=1}^{p}\langle w_\mathrm{d}\rangle \\ &= \sum_{n=1}^{N-1}\left[\left(\alpha_n + \frac{y_D}{\rho\pi r_\mathrm{d}^2}\beta_n\right)D_n + \beta_n D_n{}^2\right] \\ &\quad + 2\sum_{n=1}^{N-1}\sum_{m=n+1}^{N}\left[\beta_{nm}e^{-(m-n)(a+c)\Delta T}\right]D_n D_m\end{aligned} \quad \text{(SI-3)}$$

where

$$D_n = \langle z_n\rangle = \int_0^\infty z_n f_z(z_n)\mathrm{d}z_n\,, \quad \text{(SI-4)}$$

$$D_n{}^2 + \gamma D_n = \langle z_n\rangle^2 + \frac{y_D}{\rho\pi r_\mathrm{d}^2}\langle z_n\rangle = \int_0^\infty z_n{}^2 f_z(z_n)\mathrm{d}z_n\,, \quad \text{(SI-5)}$$

$$\alpha_n = Ap\langle G_n\rangle = Ap\int_0^\infty g_n f_g(g_n)\mathrm{d}g_n\,, \quad \text{(SI-6)}$$

$$\beta_n = B'\langle G_n\rangle^2\Phi_n = \frac{B}{p}\langle G_n{}^2\rangle = Bp\int_0^\infty g_n{}^2 f_g(g_n)\mathrm{d}g_n\,, \quad \text{(SI-7)}$$

$$\beta_{nm} = B'\langle G_n\rangle\langle G_m\rangle = Bp\int_0^\infty g_n f_g(g_n)\mathrm{d}g_n \int_0^\infty g_m f_g(g_m)\mathrm{d}g_m. \quad \text{(SI-8)}$$

Here, p is the average number of domains per cell nucleus; $f_z(z_n)$ is the probability density of the specific

energy for each period; γ represents the radiation quality including the microdosimetric parameter y_D [keV/μm]; $f_g(g_n)$ is the probability density of the domain having a DNA amount g_n per domain, and $B'\equiv B/p$ and $\Phi_n=\langle g_n^2\rangle/\langle g_n\rangle^2$ (dimensionless parameter). Assuming that the DNA amount per nucleus does not change within a short period of irradiation time (i.e., $\alpha_n=\alpha_0$=constant and $\beta_n=\beta_{nm}=\beta_0$=constant) in the constant absorbed dose rate $\dot{D}$ during irradiation time T [h] ($\langle z_1\rangle=\langle z_2\rangle=, \ldots, =\langle z_N\rangle=\dot{D}\Delta T$), we have

$$\begin{aligned}\langle w\rangle_{\mathrm{T}} &= \sum_{n=1}^{N}\left[(\alpha_0+\gamma\beta_0)\dot{D}\Delta T+\beta_0\left(\dot{D}\Delta T\right)^2\right] \\ &\quad +2\beta_0\sum_{n=1}^{N-1}\sum_{m=n+1}^{N}\left[e^{-(m-n)(a+c)\Delta T}\right]\left(\dot{D}\Delta T\right)^2 \qquad \text{(SI-9)} \\ &= -\ln S,\end{aligned}$$

Taking the limit of N to infinity and assuming that the number of LLs per nucleus follows the Poisson distribution in the same manner as the hit probability in the Linear-Quadratic (LQ) model (*1*), Eq. AI-8 is transformed as follows:

$$\begin{aligned}\lim_{N\to\infty}(-\ln S) &= (\alpha_0+\gamma\beta_0)D+\frac{2\beta_0}{(a+c)^2T^2}\left[(a+c)T+e^{-(a+c)T}-1\right]D^2 \\ &= (\alpha_0+\gamma\beta_0)D+F\beta_0 D^2 \qquad \text{(SI-10)} \\ &= -\ln S.\end{aligned}$$

As described in the manuscript, the Lea-Catcheside time factor F[1] is included in Eq. (SI-10). Relation between irradiation time T [h] and the time factor F characterizes the cell-specific dose-rate effects on dose-response curve and linearity of the curve in high-dose range. Figure S1 shows the examples of the time factor and dose-rate effects on cell survival curve.

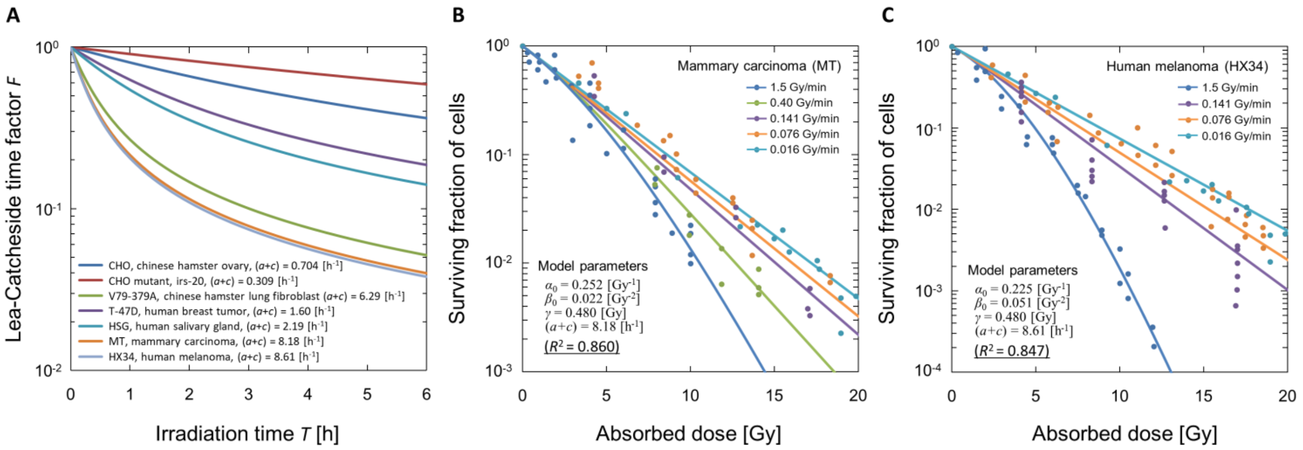

Fig. S1. Model performance of dose-rate effects: (A) for the relationship between irradiation time T [h] and the Lea-Catcheside time factor F in various cells of CHO, irs-20, V79-379A, T-47D, HSG, MT and HX3; (B) and (C) for dose-response curve for mammary carcinoma and human melanoma for various dose rates, respectively. The $(a+c)$ values as SLD repair rate were taken from the previous reports[2-4] and fitting experimental data in this study. In Figs. S1(B) and S1(C), the DNA-targeted modelling in the present model was compared with the experimental data reported by Trevor *et al* [5]. The lines and symbols represent the results predicted by the model and experimental data, respectively.

II. *Procedure of maximum likelihood method with a Monte Carlo technique*

The response parameters for signals, DSB kinetics and cell survival in this manuscript were determined by a maximum likelihood method with a Monte Carlo technique. We used a following formula as a likelihood function.

$$\ell(d,\theta) = \prod_{i=1}^{N} [\ell(d_i,\theta)]$$
$$= \prod_{i=1}^{N} \left\{ \frac{1}{\sqrt{2\pi\sigma_i^2}} e^{-\frac{(Mod_i - Exp_i)^2}{2\sigma_i^2}} \right\}, \quad \text{(SI-11)}$$

where $\ell(d,\theta)$ is the likelihood function , N is the number of experimental data, σ is the standard deviation of experimental value, *Exp* stands for the experimental value and *Mod* for the value calculated by the model. Based on Eq. (SI-11) and the algorithm illustrated in Fig. S2, we determined the set of model parameters for describing cell responses of signal, DSB kinetics and cell survival.

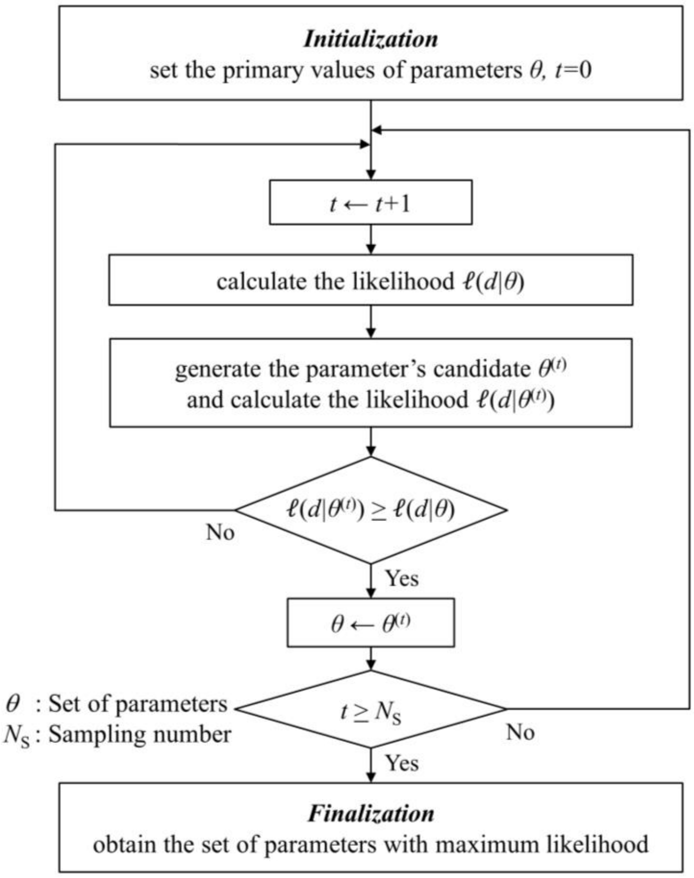

Fig. S2. Algorithm of the maximum likelihood method used in this study. We assumed that experimental value follows normal distribution, and the likelihood was calculated by using the likelihood function by Eq. (SI-11). In this study, we set that the sampling number Ns is equal to 10^7.

III. *Evaluation of log of cell surviving fraction per dose by IMK model*

As described in the results, we compared the log of cell survival (ln S) by the IMK model with experimental

data as a function of absorbed dose. In addition, here we show the log of cell survival per absorbed dose (–ln *S*/*D*) by the IMK model. The log of cell survival per dose means the average number of lethal lesions per cell induced per unit absorbed dose in this model. The survival rate in this way enables us to evaluate a more realistic appreciation of the dose-response curve than that with the usual semi-log plot of surviving fraction versus dose. By using the experimental data and the curve shown in Fig. 2(C) and 2(D), we checked the model performance about the low-dose hyper-radiosensitivity (HRS) again. Figure S3 shows the log of cell survival per dose versus dose in V79-379A and T-47D cell lines. In this figure, the curve by the IMK model considering the DNA-targeted effects (TEs) and intercellular communication (IC) agrees much better with the experimental data than that by the MK model considering only the DNA-TEs in terms of R^2 value.

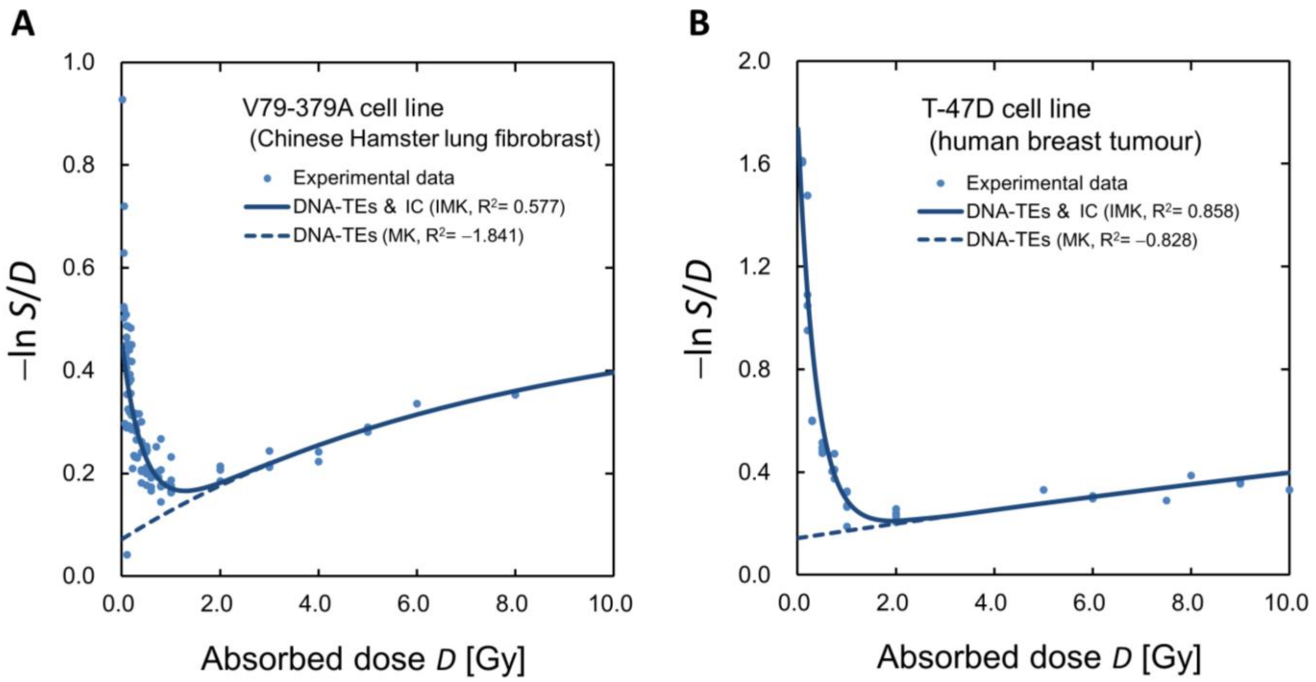

Fig. S3. Log of cell survival per dose as a function of dose in comparison with the experimental data summarized in Figs. 4(C) and 4(D), and the prediction curve by the present model: (A) for the Chinese Hamster lung fibroblast V79-379A cell line and (B) for the human breast tumour T-47D cell line. The line and symbol represent the curve by the model and experimental data, respectively. The R^2 value was calculated by Eq. (27).

IV. *Similarities and differences compared to previous models*

In the present model, the LQ relation is defined as an alternative function to the threshold-like expression or multi-targeted theory. Kundrát *et al*. modelled the mutation induction of bystander effects according to the Gompertz law[6]. Contrary to their study, in our study there is little discussion on the induction of mutation with the bystander effect. Nevertheless, assuming that the damage induction in non-hit cells is proportional to signal concentration, our model well reproduces the kinetic curves of signals and DSB number as shown in Fig. 2(B). Although further investigations are necessary for evaluating the signal-induced mutation (damage) mechanism, it is likely that the damage induction by NTEs is proportional to signal concentration as well as

the target activation number in the hit cells for emitting signals

The present model considering the signal-induced damage in non-hit cells is similar to the Bystander and Direct (BaD) model[7] or the ByStander Diffusion Modelling (BSDM) model[8]. In the BSDM model, the number of bystander cells killed is equivalent to the fraction of non-hit cells receiving specific cell-to-cell signals from a directly inactivated cell. By taking account of the fraction of non-hit cells, the IMK model can exhibit clearly the characteristics of a low-dose HRS and an involvement of the repair efficiency (Figs. 2(C) and 2(D) and Fig. 4).

REFERENCES

1. Brenner DJ. The linear-quadratic model is an appropriate methodology for determining isoeffective doses at large doses per fraction. *Semin. Radiat. Oncol.* 18: 234–239 (2008).
2. Hawkins RB. A microdosimetric-kinetic model of cell death from exposure to ionizing radiation of any LET, with experimental and clinical applications. *Int. J. Radiat. Biol.* 69:739–55 (1996).
3. Inaniwa T, Suzuki M, Furukawa T, Kase Y, Kanematsu N et al. Effects of dose-delivery time structure on biological effectiveness for therapeutic carbon-ion beams evaluated with microdosimetric kinetic model. *Radiat. Res.* 180:44–59 (2013).
4. Matsuya Y, Tsutsumi K, Sasaki K, Date H. Evaluation of the cell survival curve under radiation exposure based on the kinetics of lesions in relation to dose-delivery time. *J. Radiat. Res.* 56:90-99 (2015).
5. Trevor CS, John JE, John HP, G. Gordon S. Split-dose and low dose-rate recovery in four experimental tumour systems. *Int. J. Radiat. Biol.*, 52(1): 157-170 (1987).
6. Kundrát P, Friedland W. Friedland W. Mechanistic modelling of radiation-induced bystander effects. *Radiat. Prot. Dos.* 166:148-151(2015).
7. Brenner DJ, Little JB, Sachs RK. The bystander effect in radiation oncogenesis: II. A quantitative model. *Radiat. Res.* 155(3): 402-408 (2001).
8. Nikjoo H, Khvostunov IK. Biophysical model of the radiation-induced bystander effect. *Int. J. Radiat. Biol.* 79(1): 43-52 (2003).
